# Poor physical activity levels and cardiorespiratory fitness among patients with childhood-onset takayasu arteritis in remission: a cross‐sectional, multicenter study

**DOI:** 10.1186/s12969-021-00519-z

**Published:** 2021-03-22

**Authors:** Camilla Astley, Saulo Gil, Gleice Clemente, Maria Teresa Terreri, Clovis Artur Silva, Lucia Maria Arruda Campos, Nadia Emi Aikawa, Ana Lúcia de Sá Pinto, Rosa Maria R. Pereira, Hamilton Roschel, Bruno Gualano

**Affiliations:** 1 Applied Physiology & Nutrition Research Group, Laboratory of Assessment and Conditioning in Rheumatology, Faculdade de Medicina FMUSP, Universidade de Sao Paulo, SP Sao Paulo, Brazil; 2 Rheumatology Division, Faculdade de Medicina FMUSP, Universidade de Sao Paulo, Sao Paulo, SP Brazil; 3 Division of Paediatric Rheumatology, Department of Paediatrics, Federal University of Sao Paulo, SP Sao Paulo, Brazil; 4 Paediatric Rheumatology Unit of Children and Adolescents’ Institute, Faculdade de Medicina FMUSP, Universidade de Sao Paulo, Sao Paulo, SP Brazil

**Keywords:** Physical activity, Pediatric rheumatology, Physical fitness, Cardiovascular risk, Cardiovascular health

## Abstract

**Background:**

It is currently unknown whether patients with childhood-onset Takayasu disease (c-TA) are prone to physical inactivity and poor aerobic capacity. In this study, we assessed physical activity levels and cardiorespiratory fitness along with health-related quality of life (HRQL) and various traditional and non-traditional risk factors in patients with c-TA vs. healthy controls.

**Methods:**

c-TA patients with non-active disease (*n* = 17) and age- and sex-matched healthy controls (*n* = 17) were enrolled in the study. We assessed physical activity levels, aerobic capacity, body composition, systemic inflammation, cardiometabolic markers, disease-related parameters, and HRQL.

**Results:**

c-TA patients showed greater time spent in sedentary behavior (*P =* 0.010), and lower moderate-to-vigorous physical activity (*P* > 0.001) and lower step counts per day (*P* > 0.001). VO_2peak_ (*P* < 0.001) and chronotropic response (*P =* 0.016) were significantly lower in patients with c-TA and they had worse HRQL in physical domain (*P* < 0.001), lower bone mineral content and density, and higher insulin levels vs. healthy controls (all *P* ≤ 0.05).

**Conclusions:**

c-TA patients exhibited reduced physical activity levels and aerobic capacity, worse cardiometabolic risk factors and HRQL parameter compared with healthy peers. Physical inactivity and aerobic deconditioning emerge as potentially novel risk factors for c-TA. The role of physical activity interventions in preventing poor outcomes and improving HRQL in c-TA remains to be explored.

## Background

Childhood-onset Takayasu Arteritis (c-TA) is a very rare, granulomatous, chronic large-vessel vasculitis that involves mostly the aorta and its major branches [1]. The main pathophysiological features of this disease include increased expression of interleukin 1 (IL-1) and IL-6 in aortic tissues, and up-regulated tumor necrosis factor alpha (TNF-α) from peripheral blood mononuclear cells [2, 3]. Importantly, c-TA shows a high mortality rate (~ 35 %) [4–6] that is related to cardiovascular diseases, such as arterial occlusions and stenosis, ischemia or aneurysm formation [7].

c-TA patients show cardiometabolic abnormalities including increased cholesterol and triglycerides levels, and decreased high-density lipoprotein [HDL] cholesterol levels, impaired insulin sensitivity, and an exacerbated inflammation [4, 5]. All of these risk factors could be aggravated by an inactive lifestyle, which, in turn, could further worse clinical symptoms, traditional (e.g., diabetes, hyperlipidemia, and hypertension) and non-traditional cardiometabolic risk factors (e.g., inflammation, oxidative stress, endothelial dysfunction), physical capacity, and overall health-related quality of life (HRQL), as seen in other pediatric rheumatic diseases [8–10]. Whether due to direct limitations imposed by the disease in the cardiovascular involvement, and other indirect factors (e.g., superprotection by parents and health practitioners, low self-efficacy and, social isolation), one could surmise that patients with c-TA could also experience low physical activity levels and decreased aerobic conditioning, however, supporting evidence remains scarce.

Thus, we assessed physical activity levels and cardiorespiratory fitness along with HRQL parameters and various traditional and non-traditional cardiovascular risk factors related to physical activity and physical capacity among patients with c-TA.

## Methods

### Study design and patients

This was a multicenter, cross-sectional study conducted in Sao Paulo, SP, Brazil, between November 2017 and March 2019, as a part of a multicenter, randomized controlled trial aimed to test the safety and efficacy of an exercise training program in patients with c-TA (NCT03494062). The study was approved by the local ethics committees and all the procedures were in accordance with the recommendations of the Helsinki Declaration. The participants and their parents provided written informed consent before entering the study.

c-TA patients were recruited from the Division of Rheumatology of the School of Medicine and the Pediatric Rheumatology Unit of the Children and Adolescents’ Institute of the University of Sao Paulo, and from the Division of Pediatric Rheumatology of the Federal University of Sao Paulo. Inclusion criteria were: patients who fulfilled the current classification for c-TA [11] aged between 12 and 25 years old. Exclusion criteria were: pregnancy, heart failure, renal failure, cardiac, pulmonary or musculoskeletal disorders that precluded exercise training, the Pediatric Vasculitis Activity Score (PVAS) > 1 or greater [12] and acute infection in the last 30 days. Seventeen age- and sex-matched healthy controls (CTRL) were either friends referred by the patients or recruited from the Outpatient Clinics of Exercise and Sports Medicine (Clinical Hospital of the School of Medicine of the University of Sao Paulo), which follows healthy children and adolescents.

### Physical activity levels

Physical activity was objectively measured using Actigraph GT3X accelerometers. All participants were instructed to wear the accelerometer during waking hours, except when bathing or swimming, for 7 consecutive days. All participants accumulated at least 10 h of valid activity recordings per day for at least 4 days. The accelerometer was worn on an elastic belt at the waistline on the right side of the hip. Participants were instructed to complete a daily time diary to record when the device was worn and removed to ensure data accuracy (e.g., to distinguish sedentary time from non-wear time). Data were exported from the device every 15-seconds for children and adolescent and, 60-seconds for adults using ActiLife 6 software. Non-wear time was defined as a minimum of 60 min of continuous zero counts and days with at least 600 min of wear time were considered valid [13]. Freedson cut points were used to define epochs for patients with c-TA aged ≥ 18 years: sedentary time (< 100 counts/minute), light-intensity physical activity (≥ 100 to < 1,952 counts/minute), and moderate-to-vigorous physical activity (MVPA) (≥ 1,952 counts/minute) [13]. Evenson cut points were used to define epochs for patients with c-TA aged < 18 years: sedentary time (< 100 counts/minute), light-intensity physical activity (≥ 100 to < 2,296 counts/minute), and MVPA (≥ 2,296 counts/minute) [14]. All participants had valid accelerometer data (a minimum of any four valid days). Data are shown as minutes/day in each domain of intensity.

### Cardiorespiratory fitness test

Maximum graded exercise tests were performed on a treadmill (Centurion 200, Micromed), with increments in velocity or grade at every minute until volitional exhaustion. Heart rate (HR) was continuously recorded at rest (HR_rest_), during exercise and recovery using a 12-lead ECG (ErgoPC Elite, Micromed, Brazil). Resting HR (HR_rest_) was measured immediately before the test and HR_peak_ was measured at the end of the test with the subjects standing on the treadmill. Oxygen uptake (VO_2_) and carbon dioxide output (VCO_2_) were obtained through breath-by-breath sampling and expressed as a 30-second average using an indirect calorimetric system (Cortex, Metalyzer IIIB). The test was considered maximal when one of the following criteria was met: VO_2plateau_ (i.e., < 150 ml/min increase between two consecutive stages); respiratory exchange ratio value above 1.10; heart rate no less than 10 beats below age-predicted maximal heart rate. Heart rate was continuously recorded at rest and during exercise and recovery. Peak oxygen consumption (VO_2peak_) and ventilatory thresholds were determined following previous description [14]. Chronotropic response was calculated through the following formula: ([HR_peak_-HR_rest_/220-age-HR_rest_] x 100).

### Disease and healthy‐related quality of life parameters

Age at disease onset, time since diagnosis and current medications were obtained through review of medical records and interviews. Two physicians blinded to the intervention performed the clinical assessments using the following tools: Indian Takayasu Arteritis Clinical Activity Score (ITAS2010) [15] and Pediatric Vasculitis Activity Score (PVAS) [12]. General HRQL was also assessed through SF-36, which is a multi-purpose, short-form health survey with 36 questions. It yields a profile of functional health and well-being scores as well as psychometrically-based physical and mental health summary measures, with the higher total scores indicating better health condition [16].

### Bone mineral density and body composition

Participants underwent a dual-energy x-ray absorptiometry (DXA) scan (GE Healthcare®, WI, USA) to quantify bone mineral density (BMD) at lumbar spine (L1-L4), total hip and total body; bone mineral content (BMC); fat mass; percentage fat mass; lean mass Visceral adipose tissue were also measured by DXA using CoreScan™ software (enCORE version 17). All DXA measurements were carried out by the same trained technician.

### Blood sampling

After 12-hour overnight fasting, 40 mL of blood was extracted into vacutainer tubes and stored for subsequent analysis of fasting glucose and insulin, blood lipids, total cholesterol, triglycerides, anti- and pro-inflammatory biomarkers, and angiogenesis markers.

IFN-γ, IL-10, IL-12p70, IL-1ra, IL-1β, IL-6, TNF-α, VEGF and PDGF were analyzed using Milliplex MAP Human Cytokine/Chemokine Magnetic Bead Panel (Millipore Corp., Billerica, MA), following the manufacturer’s instructions.

### Statistical analysis

Data were presented as mean/median, standard deviation (SD) or interquartile range (IQR 25–75), between-group difference and 95 % confidence interval (95 %CI), unless otherwise indicated. Normality data was assessed by Shapiro-Wilk test. Two-tailed unpaired T-test (for normally distributed dependent variables) or Mann-Whitney U test (for non-normally distributed dependent variables) was used to compare patients with c-TA vs. controls. Data analysis were performed using Statistical Package for Social Sciences (SPSS), version 17.0 for Windows. Significance level was set at *P* ≤ 0.05.

## Results

Seventeen patients with c-TA (64.7 % females) aged 12–25 years, with median disease duration of 9.5 years were recruited. All patients fulfilled the classification for c-TA [11] and all patients had inactive disease based on disease activity scores previously described [12, 15]. Table 1 illustrates demographic, current clinical treatment, laboratory and disease-related parameters patients’ data. Groups were comparable regarding age, gender, weight, and body mass index (BMI) (all *P* > 0.05).

**Table 1:** Baseline, demographic and disease-related parameters in childhood-onset Takayasu arteritis (c-TA) and healthy control group (CTRL).

	c-TA (*n* = 17)	CTRL (*n* = 17)
*Demographic parameters*		
Age (years)	18.4 (19) ± 3.44 (12–20)	18.5 (20) ± 3.50 (12–21)
Sex, female, n (%)	11 (64.7)	11 (64.7)
Height (m)	1.60 (1.57) ± 0.11 (1.46–1.64)	1.65 (1.62) ± 0.12 (1.40–1.74)
Body weight (kg)	56.1 (54.8) ± 10.6 (40.6–63.8)	60.6 (62.0) ± 13.3 (31.2–71.0)
BMI (kg/m^2^)	17.4 (17.3) ± 2.68 (13.4–18.8)	18.2 (18.5) ± 3.39 (11.0-20.4)
Disease duration (years)	9.48 (10.1) ± 4.16 (7.07–11.8)	NA
Age at the disease onset (years)	9.25 (9.50) ± 4.62 (6.75-13.0)	NA
*TA classification n, (%)*		
Type I	1 (5.88)	NA
Type IIa	1 (5.88)	NA
Type III	2 (11.7)	NA
Type IV	4 (23.5)	NA
Type V	9 (52.9)	NA
Arterial hypertension, n (%)	10 (58.8)	NA
Aortic stenosis, n (%)	9 (52.9)	NA
*Biological therapy, n (%)*		
Infliximab	5 (29.4)	NA
*Non-biological therapy, n (%)*		
Methotrexate	8 (47.0)	NA
Leflunomide	5 (29.4)	NA
Prednisone	4 (23.5)	NA
Enalapril	5 (29.4)	NA
Amlodipine	3 (17.2)	NA
*Disease score*		
ITAS score	0.0 (0.0–0.0)	NA
PVAS score	0.0 (0.0–1.0)	NA

Data are expressed as mean (median) ± SD (IQR 25–75) or n (%). *BMI* (body mass index), *ITAS* (indian takayasu’s arteritis activity score 2010), *PVAS* (Pediatric Vasculitis Activity Score), *NA* (not applicable).

**Fig. 1:**
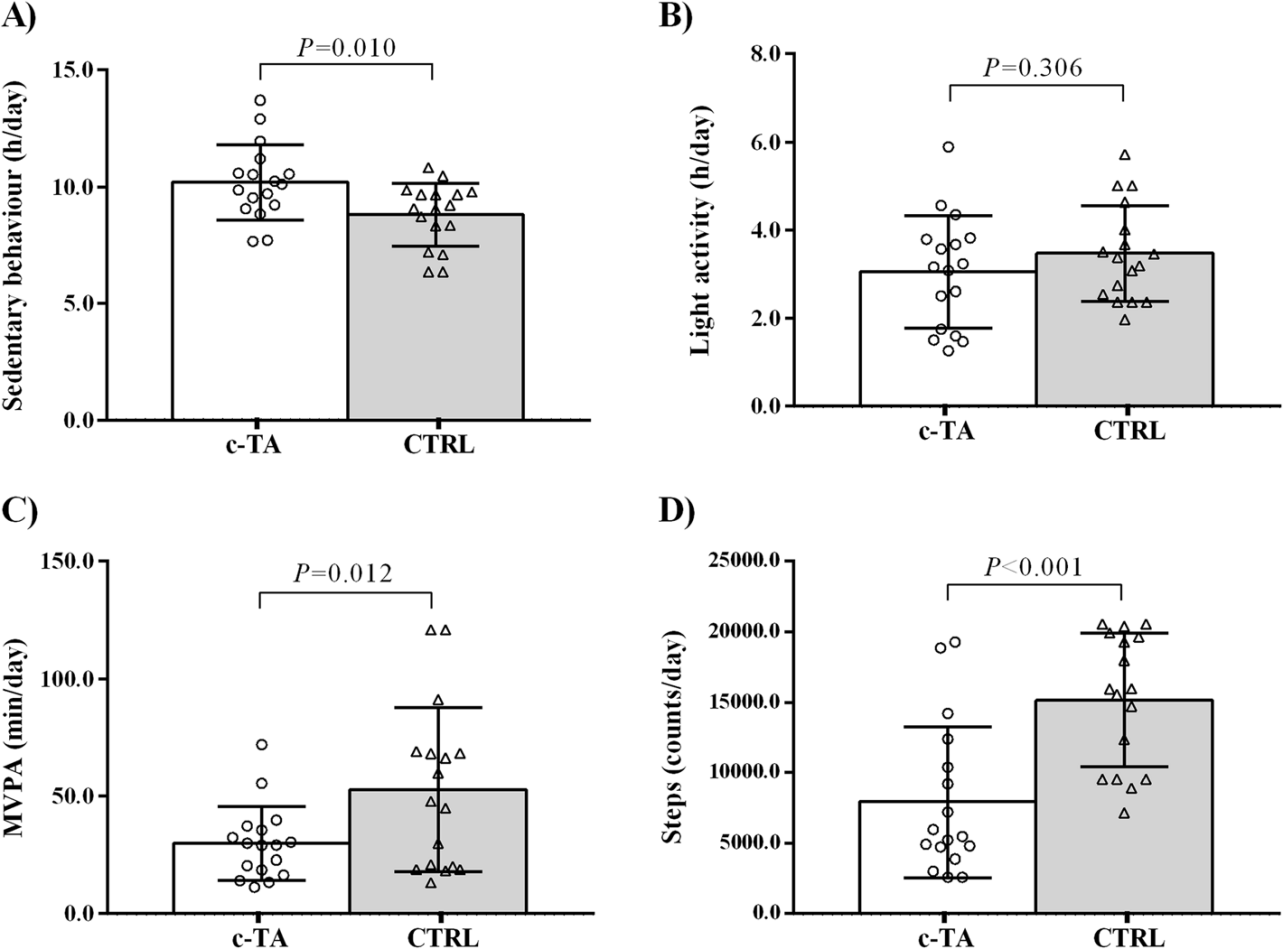
Objectively-measured physical activity in patients with childhood-onset Takayasu Arteritis (c-TA) and healthy controls (CTRL). Panel (**a**) Sedentary behavior; Panel (**b**) Light activity; Panel (**c**) Moderate-to-vigorous physical activity (MVPA); Panel (**d**) Steps per day

**Fig. 2:**
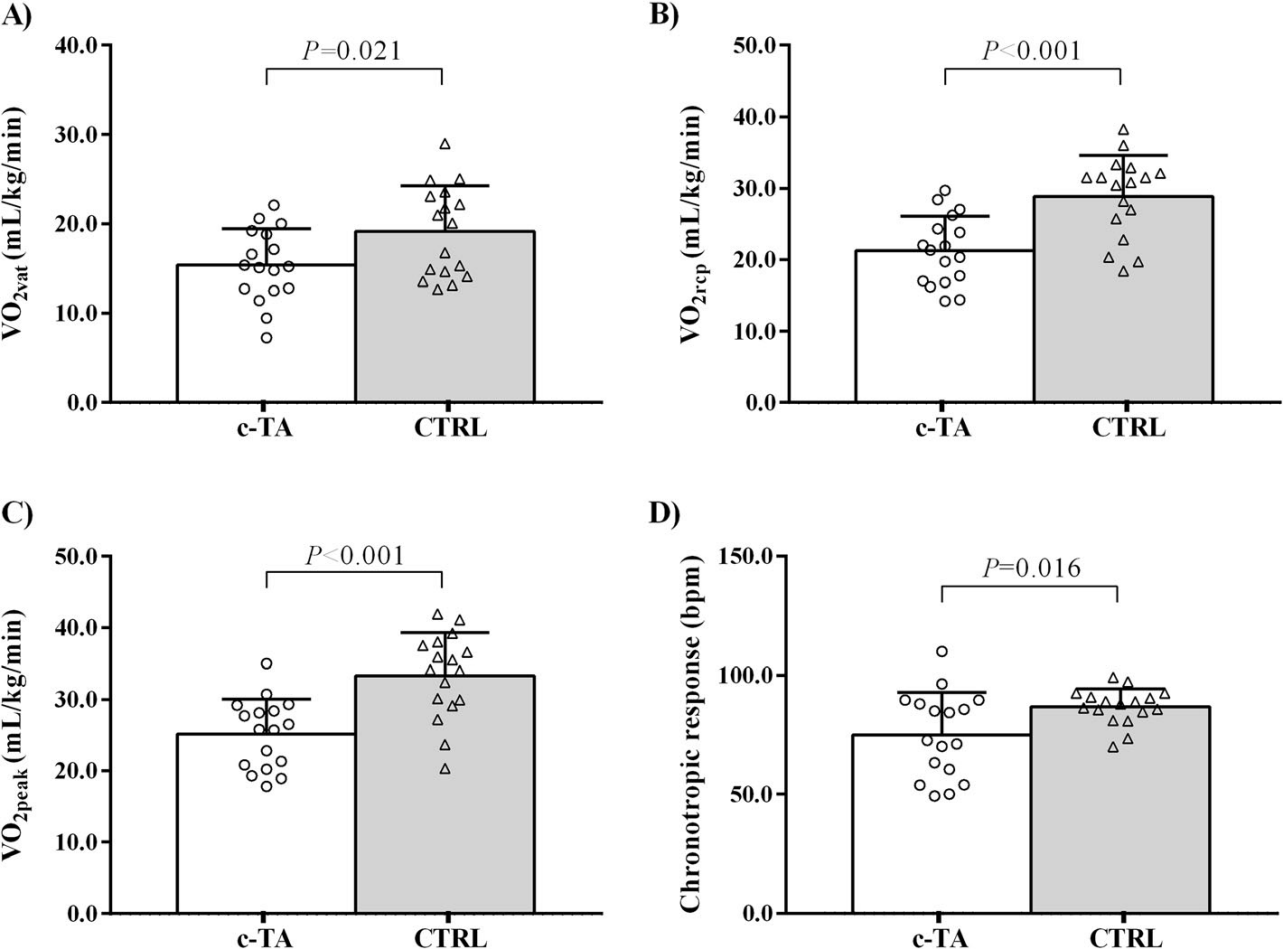
Cardiorespiratory measures in patients with childhood-onset Takayasu Arteritis (c-TA) and healthy controls (CTRL). Panel (**a**) Ventilatory threshold (VO_2vat_); Panel (**b**) Respiratory compensation point (VO_2rcp_); Panel (**c**) Peak oxygen uptake (VO_2peak_); Panel (**d**) chronotropic response

Physical activity and aerobic capacity data are shown in Figs. 1 and 2. c-TA patients exhibited greater time spent in sedentary behavior (hours per day) (*P =* 0.010), significantly lower moderate-to-vigorous physical activity (MVPA) (minutes per day) (*P* = 0.012) and step counts (*P* < 0.001) as compared to CTRL. c-TA patients also had lower VO_2vat_ (*P* = 0.021), VO_2rcp_ (*P* < 0.001), VO_2peak_ (*P* < 0.001) and chronotropic response (*P =* 0.016) vs. CTRL.

**Fig. 3:**
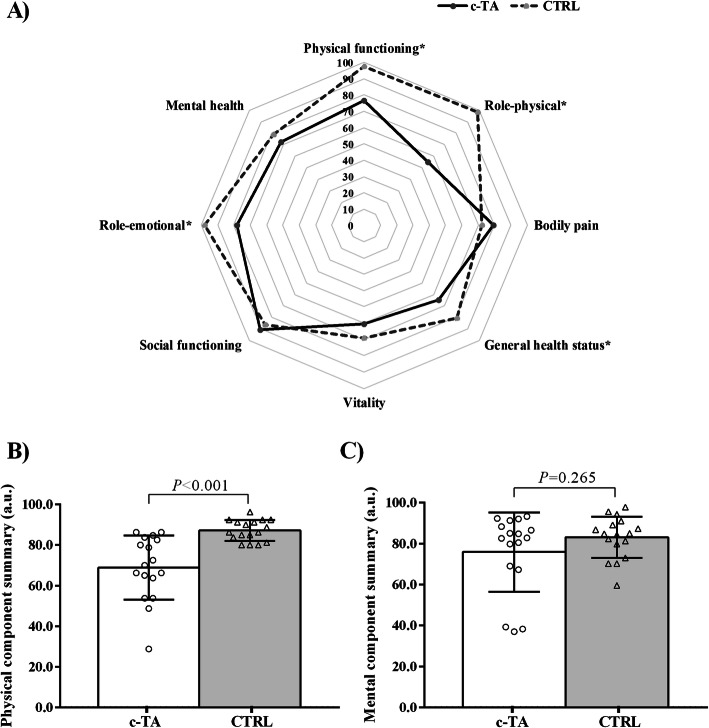
Healthy-related quality of life in patients with childhood-onset Takayasu Arteritis (c-TA) and healthy controls (CTRL). Panel (**a**) Short Form-36 health survey domains between-group; **b** Physical health domain; Panel (**c**) Mental health domain

HRQL parameters are depicted in Fig. 3. c-TA patients had worse HRQL regarding the physical domain (*P* < 0.001) compared with CTRL. As for SF-36 subscales, functional capacity (*P* = 0.021), physical appearance (*P* < 0.001), and general health status (*P* < 0.001) were reduced in patients with c-TA in comparison with CTRL. Emotional aspect tended to be lower in c-TA vs. CTRL (*P* = 0.059).

Bone mineral density and body composition measurements, systemic inflammation, and cardiometabolic risk factors are presented in Table 2. c-TA patients had significantly lower bone BMD at L1-L4 (*P* = 0.025), BMC total body (*P* = 0.023) and BMD total body (*P* = 0.032) than CTRL. In addition, patients exhibited significantly greater percent fat mass and lower lean mass (*P* = 0.024 and *P* = 0.029, respectively), when compared to controls. Inflammatory and angiogenesis markers did not differ between groups. However, patients with c-TA displayed worse insulin sensitivity, based on insulin fasting (*P =* 0.050), HOMA-IR (*P* = 0.033) and lower HDL cholesterol levels (*P* = 0.017) vs. CTRL.

**Table 2:** Body composition, systemic inflammation and cardiometabolic risk factors between childhood-onset Takayasu Arteritis patients (c-TA) and healthy controls (CTRL).

Characteristics	c-TA (*n* = 17)	CRTL (*n* = 17)	Between-group difference (95 % CI)	*P*
*DXA measurements*				
BMD (L1-L4) (g/cm^2^)	0.89 ± 0.16	1.02 ± 0.16	-0.13 (-0.01 to -0.24)	0.025
BMD total hip (g/cm^2^)	0.89 ± 0.17	0.99 ± 0.15	-0.10 (0.01 to -0.21)	0.080
BMC total body (kg)	1.86 ± 0.48	2.28 ± 0.58	-0.42 (-0.04 to -0.79)	0.023
BMD total body (kg)	1.03 ± 0.12	1.13 ± 0.13	-0.10 (-0.01 to -0.18)	0.032
Lean mass + BMC (kg)	39.6 ± 9.03	49.0 ± 13.4	-9.40 (-1.41 to -17.3)	0.028
Lean mass (kg)	37.9 ± 8.69	45.3 ± 11.2	-7.40 (-0.39 to -14.4)	0.029
Percent fat mass (kg)	30.1 ± 8.30	23.2 ± 7.80	6.90 (1.27 to 12.5)	0.024
Visceral fat (kg)	2.75 ± 1.30	2.14 ± 0.85	0.61 (-0.15 to 1.37)	0.201
*Systemic inflammation*				
C-reactive protein (mg/L)	4.84 ± 6.63	2.30 ± 1.64	2.54 (-0.83 to 5.91)	0.135
ESR (mm/1st hour)	9.18 ± 9.22	4.89 ± 2.24	4.29 (-0.39 to 8.97)	0.072
IFN-γ (mg/L)	7.70 ± 13.1	1.70 ± 0.90	6.00 (-0.48 to 12.4)	0.078
IL-10 (pg/mL)	14.8 ± 16.0	7.70 ± 3.77	7.10 (-1.02 to 15.2)	0.081
IL-12p70 (pg/mL)	5.46 ± 3.76	4.74 ± 1.90	0.72 (2.80 to -1.36)	0.488
IL-1ra (pg/mL)	123.6 ± 236.3	34.4 ± 15.6	89.2 (-27.7 to 206.1)	0.126
IL1-β (pg/mL)	2.55 ± 1.25	2.16 ± 0.40	0.39 (-0.25 to 1.03)	0.233
IL-6 (pg/mL)	21.7 ± 32.3	15.2 ± 28.4	6.50 (-14.7 to 27.7)	0.542
TNF-α (pg/mL)	15.1 ± 7.27	13.2 ± 6.60	1.90 (-2.95 to 6.75)	0.443
VEGF (pg/mL)	2.86 ± 4.89	1.67 ± 0.60	1.19 (-1.24 to 3.62)	0.337
PDGF (pg/mL)	561.4 ± 1273.8	766.1 ± 1529.3	-204.7 (778.2 to -1187.6)	0.667
*Cardiometabolic risk factors*				
SBP (mmHg)	110.9 ± 15.3	104.8 ± 8.1	6.10 (-2.45 to 14.6)	0.225
DBP (mmHg)	64.4 ± 9.30	68.3 ± 8.20	-3.90 (2.23 to -10.0)	0.251
Glucose fasting (mg/dL)	83.1 ± 8.70	82.2 ± 4.55	0.90 (-3.95 to 5.75)	0.723
Insulin fasting (mg/dL)	13.9 ± 9.85	8.37 ± 3.80	5.53 (0.31 to 10.7)	0.050
HOMA-IR	2.88 ± 2.11	1.67 ± 0.78	1.21 (0.09 to 2.32)	0.033
Total cholesterol (mg/dL)	151.2 ± 19.5	156.2 ± 18.8	-5.00 (8.38 to -18.4)	0.479
HDL cholesterol (mg/dL)	53.4 ± 13.6	66.1 ± 14.1	-12.7 (-3.02 to -22.3)	0.017
LDL cholesterol (mg/dL)	82.0 ± 16.9	75.6 ± 13.4	6.40 (-4.25 to 17.0)	0.271
VLDL cholesterol (mg/dL)	15.8 ± 2.98	14.6 ± 3.02	1.20 (-0.89 to 3.29)	0.262
Triglycerides (mg/dL)	71.2 ± 25.1	60.5 ± 14.9	10.7 (-3.72 to 25.1)	0.139

Values are means ± SD. *DXA* (dual-energy x-ray absorptiometry), *BMD* (bone mineral density), *BMC* (bone mineral content), *ESR* (erythrocyte sedimentation rate), *IFN-γ* (interferon gamma), *IL-10* (interleukin 10), *IL-12p70* (interleukin 12p70), *IL-1ra* (interleukin 1 receptor antagonist), *IL-1β* (interleukin 1 beta), *IL-6* (interleukin 6), *TNF-α* (tumor necrosis factor alfa), *VEGF* (vascular endothelial growth factor), *PDGF* (platelet-derived growth factor), *SBP* (systolic blood pressure), *DBP* (diastolic blood pressure), *HOMA-IR* (homeostatic model assessment for insulin resistance), *HDL* (high-density lipoprotein), *LDL* (low-density lipoprotein), *VLDL* (very-low-density lipoprotein),

## Discussion

We described, for the first time, physical activity levels and aerobic conditioning and multiple traditional and non-traditional cardiovascular risk factors associated with these variables among patients with c-TA. Confirming our hypothesis, patients with c-TA exhibited lower MVPA levels and increased sedentary behavior (step per day), decreased aerobic capacity (VO2_vat_, VO2_rcp_ and VO2_peak_) and decreased autonomic function (chronotropic response) when compared to healthy peers. c-TA patients also showed poorer health-related quality of life and worse body composition (BMD and lean mass) and cardiometabolic parameters (insulin sensitivity surrogates and HDL cholesterol).

TA is the most common form of large-vessel vasculitis in children and adolescents that predisposes patients to a high risk of mortality and a variety of comorbidities linked to the cardiovascular system [17]. We [18, 19] and others [20–22] have shown that patients with pediatric rheumatic diseases are often hypoactive. WHO recommends that children and adolescents aged 5 to 17 should engage in at least 60 min of MVPA daily, whereas adults aged ≥ 18 years should perform at least 150 min of MVPA per week or 75 min of vigorous activity per week, or a combination of both [23]. In our sample, none of the patients met the recommendations for physical activity levels (i.e., minimum of 60 min of MVPA per day). This is in accordance with data showing a high frequency of physical inactivity among other pediatric rheumatic diseases, such as childhood-onset systemic lupus erythematosus [19], childhood-onset dermatomyositis [24] and juvenile idiopathic arthritis [25]. Of relevance, only 3 patients from CTRL achieved the guidelines; even so, CTRL group showed higher physically activity levels than c-TA, which supports the notion that hypoactivity may be a marked feature in this disease. Physical inactivity appears to be implicated in worsen clinical symptoms, increased traditional and non-traditional risks factors, and poor HRQL among pediatric rheumatic patients (for comprehensive reviews, see [8, 26, 27]). The very high proportion of physically inactive patients in the current study suggests that physical inactivity is a potentially novel risk factor for disturbances in cardiovascular, skeletal muscle and bone systems to be considered in this disease. The main factors contributing to the low physical activity levels showed by our patients could be related to the disease comorbidities and clinical features (arterial hypertension, stenosis, claudication, etc.), although other aspects could also play a role in inactivity (e.g., physical and emotional limitations imposed by the disease, parent’s superprotection, social isolation). The actual barriers for physical activity in this condition require further investigation.

Previous studies have suggested that physical inactivity can lead to decreased aerobic capacity and autonomic dysfunction, both being independent predictors of all-cause mortality and poor HRQL [9, 10, 18]. We observed that patients with c-TA displayed poor cardiorespiratory fitness and autonomic dysfunction, in consistent with data reported for other pediatric rheumatic diseases [18, 28]. To which extent these poor prognosis parameters can increase the burden of cardiovascular diseases in c-TA needs to be investigated.

c-TA patients also showed reduced total body and lumbar spine BMD, BMC and lower lean mass as compared to their heathy counterparts. Abnormalities in bone metabolism have been reported among pediatric rheumatic diseases, possibly due to the chronic inflammation and the prolonged use of glucocorticoids and/or other immunosuppressive drugs [29]. Low physical activity can also contribute to diminished bone accretion during childhood, which may result in sub-optimal bone mass in later adulthood [30]. The lack of sufficient physical activity has also a causal link with worse cardiometabolic health, including insulin resistance and dyslipidemia [31]. In the current study, we observed lower HDL cholesterol and higher insulin levels among patients with c-TA. It is also possible that hypoactivity may account for the poor quality of life (both emotional and physical aspects, see Fig. 3) reported herein. Of clinical relevance, physical activity promotion has been shown to attenuate all these poor outcomes in several pediatric rheumatic diseases [18, 32–34]. To date, however, there is no study showing the ability of physical activity interventions in yielding beneficial effects in c-TA.

This study has some limitations. First, although data were collected in three centers, the sample size was yet limited due to the very rare nature of this severe disease. Second, patients’ characteristics such as age, sex, and current treatment were relatively heterogeneous. Future studies should try to replicate these data in more homogenous samples. Third, all our patients were in disease remission and had a well-controlled disease. It is important to investigate to which extent, if any, disease activity can aggravate physical inactivity and aerobic deconditioning.

## Conclusions

In conclusion, patients with c-TA in disease remission showed reduced physical activity levels and aerobic conditioning as well as poor traditional and non-traditional cardiovascular risk factors and quality of life as compared to healthy peers. These findings reveal that physical inactivity and aerobic deconditioning are potentially novel risk factors for c-TA. The role of physical activity interventions in preventing poor outcomes and improving HRQL in c-TA remains to be explored.

## Data Availability

All data generated or analysed during this study are included in this published article.

## Abbreviations

c-TA: Childhood onset Takayasu arteritis
IL-6: Interleukin six
TNF-α: Tumor necrosis factor alpha
HDL: High-density lipoprotein
HRQL: Health-related quality of life
PVAS: The paediatric vasculitis activity score
CTRL: Control group
ITAS2010: The Indian Takayasu Arteritis Score 2010
DXA: Dual-energy x-ray absorptiometry
BMD: Bone mineral density
BMC: Bone mineral content
BMI: Body mass index
MVPA: Moderate-vigorous physical activity
